# Factors Associated with Serious Injuries among Adolescents in Ghana: Findings from 2012 Global School Health Survey

**DOI:** 10.1155/2021/6622363

**Published:** 2021-04-20

**Authors:** Martin Ackah, Mohammed Gazali Salifu, Hosea Boakye

**Affiliations:** ^1^Physiotherapy Department, Korle Bu Teaching Hospital-Ghana, Accra, Ghana; ^2^Ministry of Health, Accra, Ghana; ^3^Physiotherapy Department, LEKMA Hospital-Ghana, Accra, Ghana

## Abstract

**Introduction:**

Injuries are of public health concern and the leading cause of residual disability and death among teenagers, especially in low- and middle-income countries (LMICs). In Ghana, the burden of injury among adolescents is under-reported. Hence, the study sought to determine the prevalence of serious injuries (SI) and the potential factors influencing these injuries among school children in Ghana.

**Methods:**

This study was conducted in Ghana among Junior High School (JHS) and senior high school students (SHS) using the 2012 Global School-Based Student Health Survey (GSHS) data. The GSHS employed two-stage cluster sampling method. Serious injuries (SI) and independent factors were measured via self-administered questionnaires. Pearson chi-square test between each explanatory variable and serious injuries was conducted and the level of statistical significance was set at 5%. The significant variables from the chi-square test were selected for multiple logistic regression analysis. Multiple logistic regression was performed to estimate the adjusted odds ratio (AOR) at 95% confidence interval (CI).

**Results:**

The prevalence of SI in the past 12 months was 66% [CI=61.8–70.2] . The most common cause of SI was fall, 36%. The common types of injuries were cut/stab wounds and broken/dislocated bone. In the multiple logistic regression analysis, after controlling for other variables, educational level (AOR = 0.64, CI = 0.44–0.90, *p* < 0.015), suicidal ideation (AOR = 1.58, CI = 1.00–2.48, *p* < 0.002), suicidal attempt (AOR = 1.88, CI = 1.29–2.72, *p* < 0.001), having at least one close friend (AOR = 1.49, CI = 1.17–1.89, *p* < 0.002), school truancy (AOR = 1.66, CI = 1.31–2.09, *p* < 0.000), smoking marijuana (AOR = 2.64, CI = 1.22–5.69), and amphetamine use (AOR = 2.95, CI = 1.46–5.69) were independently associated with SI.

**Conclusion:**

The findings of the study established a high prevalence of SI among adolescents in Ghana, with cut/stab wound and broken/dislocated bone being the most reported type of injuries. This study also revealed that factors such as educational level, suicidal ideation, suicidal attempt, at least one close friend, school truancy, smoking marijuana, and amphetamine use are associated with SI among the adolescents. Therefore, pragmatic interventional programs should be targeted at these factors to curb the rate of SI among junior and senior school students.

## 1. Introduction

Globally, injuries are of public health concern and the leading cause of residual disability and death among those under 19 years, especially in low- and middle-income countries [1]. Global burden of disease estimated that 5.1 million deaths occurred from injuries in 2010, of which 12% are attributed to unintentional injuries among children and adolescents [2]. In sub-Saharan African (SSA), 250 million people are aged 10–19 years and this number is expected to increase by 24% by 2020 [3]; however, less attention is paid to adolescent injuries largely due to the greater attention devoted to widespread nutrition deficiencies and communicable diseases [4]. The estimated incidence of injuries for children is 53.1/100000 in SSA [4]. In addition, all injury-related mortalities occur globally, more than 95% occur in low- and middle-income countries with detrimental physical, psychological, and economic effects [5].

Prior epidemiological studies have indicated that injuries among adolescents have declined by 50% in developed countries such as Britain, US, Austria, and Canada over the past 30 years through “multisectored approach” to prevention [1, 6]. For instance, the prevalence of injury was approximately 21% in Europe [7], 24% in Canada [8], and 38% in China [9]. In low-income countries, such studies are mostly limited to hospital and community-based information [1] and the prevalence is relatively higher. For example, the prevalence of injuries was 62% in Ethiopia [5] and 73.6% in Nigeria [10].

In Ghana, the burden of injuries among adolescents is not well described even though it is hypothesized to be higher based on data in other LMICs. However, Gyedu et al. [11] observed that household child injury (HCI) burden in Ghana is about ten times higher than that reported from the United States [12]. In addition, factors influencing serious injuries such as psychological, personal, and school environment factors have not been extensively studied among these targeted populations (adolescents attending school).

Therefore, this study sought to assess and determine the prevalence of SI and its potential influencing factors among adolescents attending school in Ghana. This will aid in the development of preventive and monitoring strategies against serious injuries among adolescents in Ghana.

## 2. Methods and Materials

### 2.1. Study Design

The researchers utilized data from the 2012 Global School-Based Student Health Survey (GSHS), Ghana version [13]. The GSHS is a school-based survey which uses a self-administered questionnaire. The survey obtained data on health behavior and risk factors associated with the principal cause of death and mortality among school children and young-adults globally. The GSHS was carried out by the WHO with assistance from Disease Control and Prevention (CDC), Middle Tennessee State University, and the Ghana Education Service (GES). The data were obtained using a cross-sectional survey design among WHO countries which were concerned in assessing the behavioral risk factors and protective factors among junior and senior high school students. 

### 2.2. Ethical Consideration

The questionnaire was piloted before the actual data collection to ensure sufficient understanding of the survey items. The survey was approved at Middle Tennessee State University by Institutional Review Board. All ethical considerations and policies from Ghana Education Service (GES) were strictly adhered to during the survey. Permissions were also obtained from GES, heads of the selected schools, and classroom teachers. Both verbal and written assents were obtained from all the students. For minors, assent was obtained from their parents as well. The dataset is freely available for download at http://www.who.int/chp/gshs/en/.

### 2.3. Sampling

The participants were students from JHS and SHS in Ghana. A two-stage cluster sample design was used to obtain representative information of all the students in the selected schools across the country. During the first stage, schools were selected with probability proportional to enrollment size. At the second stage, classes were randomly selected and all students in the selected classes qualified to partake in the study. The overall response rate was 82% and 74% for JHS and SHS, respectively.

### 2.4. Variables

Outcome variable: the main dependent variable was the prevalence of SI among the participants. It was ascertained by a single question, “during the past 12 months, how many times were you seriously injured?” The options ranged from zero (0) times to twelve (12) or more times. The current study further dichotomized the responses. Those with zero injury were grouped as “no injury” and coded as 0 and those with at least one or more injuries as “serious injuries” coded as 1.

Explanatory variables: these variables were broadly grouped into socio-demographic factors (sex, education, age, hunger, and number of friends), psychological factors (suicidal ideation, planning, and attempt), and personal attributes (truancy, amphetamine, marijuana smoking, and alcohol use]. Explanatory variables are as shown in Table 1.

### 2.5. Data Analysis

In all analyses, sample weights were applied in order to make it generalizable to the population and further minimize bias on various trends of nonresponses. Some variables were recoded on binary scale in this study as in other existing GSHS study [14–16]. Participants aged 11 years and below were dropped from the analysis. The primary analyzes were carried out in two steps to evaluate variables that were strongly associated with SI among adolescents in Ghana. First, bivariable analyzes using Pearson chi-square were used to investigate potential relationship between the explanatory variables and SI. Variables that showed significant association (*p* < 0.05) were entered into a binary logistic regression model in the second step. Furthermore, multiple logistic regression analyses were used to assess the explanatory variables that independently predict the outcome variable (SI). The results from the multiple logistic regression analyzes were presented as adjusted odds ratio at 95% confidence interval (CI). *p* values less than 0.05 were interpreted as significant in all analyzes.

Missingness was addressed using the multiple imputation (MI) technique. The technique was applied to variables where the missing values exceed 5%. The missing data ranged from 5% to 11%. The items were missing at random (MAR). The primary sampling unit (PSU), strata, and weight were included in the imputation process as a result of the complex survey design employed during the data collection. MI was used to construct and analyze 5 multiply imputed datasets. The parameter of interest (i.e., serious injuries, amphetamine, and current smoker (marijuana)) were estimated in each imputed dataset separately and combined using Rubin's rule [17]. Imputed values compared reasonably to observed values and results using the complete case analysis which were similar to MI.

The final model goodness of fits was checked [18] using the command “syvlogitgof.” The results revealed no evidence of a lack of fit with our model in significantly predicting serious injuries.

All analyzes were carried out with STATA (Stata Statistical Software: Release 16; College Station, TX; Stata Corp LP) software and Microsoft Office 2013.

## 3. Results

### 3.1. Background Characteristics of the Adolescents in Ghana

**Figure 1:**
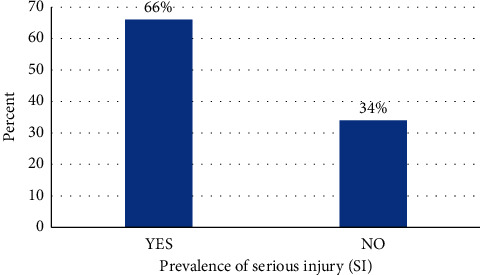
Serious injuries among adolescents attending school in Ghana.

**Figure 2:**
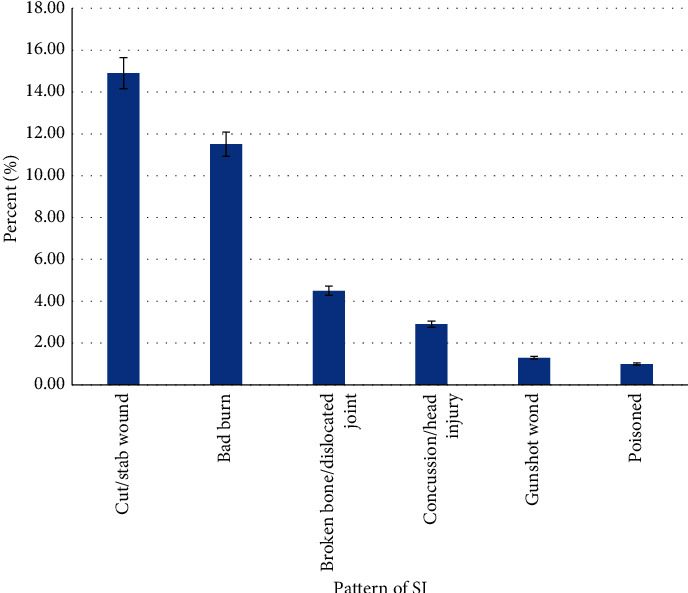
Pattern of serious injuries among adolescents in Ghana.

**Figure 3:**
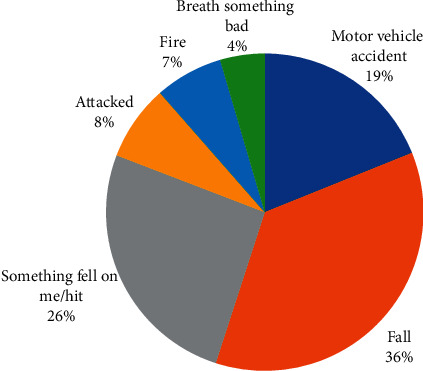
Cause of serious injuries among adolescents in Ghana.

 Out of the 3592 adolescents attending school, 53.3% were males and 43.1% were aged between 15 and 17 years. Using hunger as a proxy to socio-economic status, 14% went hungry for the past 30 days. The prevalence of suicidal ideation, suicidal planning, and suicidal attempts was 18.9%, 22.4%, and 24.7%, respectively (Table 2). The prevalence of serious injuries (SI) was 66% (CI = 61.8–70.2) in the current study (Figure 1). Amphetamine, current drinker (alcohol), and current smoker (marijuana) were 6.5%, 12.9%, and 5.0%, respectively.  Figure 2 shows the pattern of injuries among the participants; of those who had serious injuries, majority were cut/stab wound 14.9% (CI = 13.4–16.5), followed by broken bone/dislocated joint 11.5% (CI = 9.6–13.9), concussion/head injury 4.5% (3.6–5.6), bad burn 2.9% (CI = 2.3–3.8), gunshot wound 1.3% (CI = 0.8–2.1), and Poisoned 1.0% (CI = 0.5–1.9).  Figure 3 depicts the cause of serious injuries among adolescents in Ghana.

### 3.2. Distribution and Chi-Square Analysis of Serious Injuries across Demographic, Psychological, and Personal Attribute Factors

The findings of the bivariate analysis are presented in Table 3. Socio-demographic factors, educational level, being hungry, and having at least one friend were found to be significantly associated with SI. Sex was not associated with SI (*p* > 0.05). Also, psychological factors such as suicidal ideation, suicidal plan, and suicidal attempts were found to be statistically significant. Finally, school truancy, amphetamine used, and smoking marijuana were also found to be significantly associated with SI.

### 3.3. Association between the Significant Variables and Serious Injuries

The results of the binary logistic regression analysis are presented in Table 4. Sex was added to the model though it was nonsignificant in the bivariate analysis. After controlling for other variables, educational level (AOR = 0.64, CI = 0.44–0.90, *p* < 0.015), suicidal ideation (AOR = 1.58, CI = 1.00–2.48, *p* < 0.002), suicidal attempt (AOR = 1.88, CI = 1.29–2.72, *p* < 0.001), at least having one close friend (AOR = 1.49, CI = 1.17–1.89, *p* < 0.002), school truancy (AOR = 1.66, CI = 1.31–2.09, *p* < 0.000), and amphetamine use (AOR = 2.95, CI = 1.46–5.69) added significance to the model.

## 4. Discussion

The current study aimed to determine the prevalence of SI and its determinants among school children in Ghana. The prevalence of SI in this study was reported to be 66 percent. There seems to be a huge disparity in the prevalence of injuries between developed countries and that of SSA. While countries in the SSA report high prevalence of injuries, their counterparts in the developed world have appreciably low prevalence. For instance, the prevalence of injuries was approximately 21% in Europe [7], 24% in Canada [8], and 38% in China [9], while that of Nigeria [10] was 74%, Liberia [19] 72%, and Ethiopia [5] 63%. The observed disparities could be ascribed to a number of factors. Firstly, there is vast literature on awareness and prevention approaches of injuries in the developed countries but such interventions are scarce in SSA. In addition, according to Salam et al. [20], political commitment towards enactment and implementation of polices are crucial in the prevention of injuries. However, such commitment on the part of political leaders in SSA could be said to be low as compared to that of developed countries.

With regards to the pattern of injuries, cut/stab wounds and broken/dislocated bones dominated. The findings corroborate with previous studies in four Asian countries [21] and Gao et al. [9] in China, where sprain/fracture was also found to be dominant among adolescents. Furthermore, fall and motor-vehicle accident were the most common causes of injuries among these populations. This is in agreement with [22–24] and earlier hospital-based and population household-based Ghanaian studies [11, 25].

Serious injuries were more prevalent in the female population than male population (53% vs. 51%) and had 17% increased odds of sustaining SI as compared to their male counterpart. However, this was not statistically significant. This is in line with the recent cross-sectional analysis of Liberian adolescents using Global Health School Survey, which also found that sex had no significant association with serious injuries [19]. However, the current findings contradict studies in Palestine [1], Asia [21], and Yemen [23]. The current findings could be ascribed to the fact that majority of the injuries occurred at home where females tend to sustain more injuries as observed by Rommel et al. [26]. In addition, the lack of significant association maybe because intentional injuries were included and tend to affect more to female populations in some contexts.

Hunger as a proxy to socio-economic status was associated with serious injuries among adolescents in Ghana. For example, being hungry was linked with 2.6 increased odds of getting serious injuries as compared to those who were not hungry. This corroborates with Peltzer and Pengpid [27] in Malaysia, four Asian countries (i.e., Indonesia, Sri Lanka, Thailand, and Myanmar) [21], and Canada [28]. In the contrary, Jildeh et al. [1] reported lower prevalence of injuries among adolescents from lower socio-economic family in Palestine. Although socio-economic status has been associated with the development of injuries among children, there are inconsistencies across boundaries in determining which class of social status is more prone to developing injuries. Due to the above, different cultural perceptions of social-economic status could probably account for the inconsistencies.

In other dimensions, senior high school children had a decreased odd of sustaining one or multiple injuries. The possible explanation could be that most second cycle institutions in Ghana are in a “boarding-house”, hence the students' mobility is partly confined and regulated by the school authorities relative to junior high school students in the country. Likewise, SHS students' exposures to previous injuries might be influencing their current risky behaviors compared with JHS students.

Similar to prior studies, the survey found an association between psychological variables such as suicidal attempts and suicidal ideation [9, 21, 24], school truancy [24], and injuries. Therapy services in the high schools should be strengthened and be able to recognize these children in psychological distress and give their full support. In addition, parents need a close relationship with their children so that they can help their children overcome their psychological problems.

In this study, amphetamine use was independently associated with one or multiple injuries. These findings corroborate [9, 24]. A holistic public health strategy is an effective way to tackle substance usage in a school setting which should be enhanced. This can be done through education, preventive health services, social support, and healthy physical environment and establish connections with the wider community.

The current study found an association between smoking marijuana and SI. This is consistent with a previous study of Adolescent Health and Lifestyle Survey (AHLS) among 8219 adolescents aged 12–18 years [29] and study by Choi et al. [30] in South Korean. In the same vein, a dose-response association was shown between smoking and injury, with the risk of injuries greater than that of nonsmokers by 27% for low-level smokers, 37% for moderate smokers, and 71% for high-level smokers in the United States [31].

## 5. Conclusion

The findings of the study established a high prevalence of SI among adolescents in Ghana, with cut/stab wounds and broken/dislocated bone being the most reported type of injuries. This study also revealed that factors such as educational level, suicidal ideation, suicidal attempt, having at least one close friend, school truancy, smoking marijuana, and amphetamine use are associated with SI among the adolescents. Therefore, pragmatic interventional programs should be targeted at these factors to curb the rate of SI among adolescents in Ghana.

## Figures and Tables

**Table 1:** Explanatory variables data derivation from the survey.

Variable	Survey question	Coding
Age	How old are you?	0 = 12–14 yrs.
		1 = 15–17.
		2 ≥ 18 yrs.
Sex	What is your sex?	1 = male, 2 = female
Educational level	In what grade are you?	0 = JHS
		1 = SHS
Hunger	Students who went hungry most of the time or always because there was not enough food in their home during the past 30 days	Yes = 1
		No = 2
Close friends	How many close friends do you have?	0 friend = 1
		≥1 friend = 2
Suicidal ideation	During the past 12 months, did you ever seriously consider attempting suicide?	Yes = 1;
		No = 2
Suicidal plan	During the past 12 months, did you make a plan about how you would attempt suicide?	Yes = 1
		No = 2
Suicidal attempt	During the past 12 months, how many times did you actually attempt suicide?	1 = 0 times
		2 = ≥1 time
School truancy	During the past 30 days, how many days did you miss classes or school without permission?	1 = ≥1 day(s)
		2 = 0 day
Amphetamine use	During your life, how many times have you used amphetamine or methamphetamine (also called ice or yellow)	1 = ≥1 time (s)
		2 = 0 time
Current use of alcohol	During the past 30 days, how many days did you have at least one drink containing alcohol?	1 = 0 day
		2 = ≥1 day (s)
Current use of marijuana	During the past 30 days, how many times have you used marijuana (also called wee, jah, Indian hemp, ahabammono, and ganja)?	1 = 0 times
		2 = ≥1 time

**Table 2:** Background characteristics of the school-going adolescents in Ghana (*n* = 3592).

Factor	Variable	Frequency	Percent (%)
*Demographic factors*			
Sex	Male	1914	53.3
	Female	1641	45.7
	Missing	37	1.0
Educational level			
	JHS	1603	44.6
	SHS	1974	55.0
	Missing	15	0.4
Age category			
	12–14	842	23.4
	15–17	1549	43.1
	18+	1182	32.9
	Missing	19	0.5
Went hungry			
	Yes	503	14.0
	No	3078	85.7
	Missing	11	0.3
Close friends			
	0	452	12.6
	≥1	3098	86.2
	Missing	42	1.2

*Mental health factors*			
Suicide ideation	Yes	680	18.9
	No	2870	79.9
	Missing	42	1.2
Suicide planning			
	Yes	806	22.4
	No	2694	75.0
	Missing	92	2.6
Suicide attempt			
	Yes	887	24.7
	No	2666	74.2
	Missing	39	1.1

*Personal attributes*			
Truancy	Yes	1295	36.0
	No	2244	62.5
	Missing	53	1.5
Amphetamine use	Yes	232	6.5
	No	3072	85.5
	Missing	288	8.0
Current drinker (alcohol)	Yes	464	12.9
	No	2858	79.6
	Missing	270	7.5
Current smoker (marijuana)	Yes	181	5.0
	No	3259	90.7
	Missing	152	4.2

**Table 3:** Distribution of serious injuries across demographic, psychological, and personal attribute factors.

		Serious injury		Chi-square	*p* value
		Injury (%)	No injury (%)		

*Demographic factors*					
Sex					
	Male	1013 (51.3)	881 (48.7)	0.704	0.405
	Female	632 (52.9)	505 (47.1)		
Educational level					
	JHS	973 (72.7)	369 (27.3)	91.077	0.001^*∗*^
	SHS	938 (55.9)	770 (44.1)		
Age (years)	12–14	500 (70.8)	206 (29.2)	54.100	0.000^*∗*^
	15–17	855(64.8)	465 (35.2)		
	≥18	553 (56.5)	468 (43.5)		
Number of close friends					
	None	217 (60.3)	164 (39.7)	6.0147	0.013^*∗*^
	≥1	1672 (66.7)	971 (33.3)		
Hunger (economic status)					
	Low	312(75.0)	119 (25.0)	18.2257	0.006^*∗*^
	High	1602 (64.7)	1,019 (35.3)		

*Psychological factors*					
Suicidal ideation	Yes	450 (80.4)	118 (19.6)	70.4520	0.000^*∗*^
	No	1439 (62.2)	1023 (37.8)		
Suicidal planning	Yes	500 (75.5)	175 (24.5)	39.9194	0.000^*∗*^
	No	1355 (62.5)	956 (37.5)		
Suicidal attempt					
	Yes	612 (81.9)	150 (18.9)	126.0745	0.000^*∗*^
	No	1280 (60.1)	956 (39.9)		

*Personal attributes*					
Truancy	Yes	797 (75.4)	308 (24.6)	74.432	0.000^*∗*^
	No	1095 (60.2)	822 (39.8)		
Amphetamine use					
	Yes	176 (92.2)	18 (7.8)	82.2795	0.000^*∗*^
	No	1559 (62.2)	1072 (37.8)		
Current drinker					
	Yes	304 (80.9)	86 (119.1)	50.0614	0.000^*∗*^
	No	1451 (62.9)	993 (37.1)		
Current smoker (alcohol)					
	Yes	139 (93.7)	11 (6.3)	68.989	0.000^*∗*^
	No	1672 (63.3)	1109 (36.7)		

^*∗*^Significant at *p* < 0.05.

**Table 4:** Association between the significant variables and serious injuries.

Variables	Unadjusted OR 95%	*p* value	Adjusted OR 95%	*p* value
*Sex*				
Male	Ref.		Ref.	
Female	1.07 [0.91–1.25]	0.405	1.17 [0.95–1.46]	0.130

*Educational level*				
JHS	Ref.		Ref.	
SHS	0.48 [0.35–0.65]	0.000	0.64 [0.44–0.90]	0.015

*Age (year)*				
12–14	Ref.		Ref.	
15–17	0.83 [0.65–1.08]	0.161	1.13 [0.81–1.58]	0.454
18 and above	0.51 [0.39–0.66]	0.000	0.73 [0.52–1.02]	0.305

*Hunger*				
No	Ref.			
Yes	1.64 [1.17–2.33]	0.007	1.37 [1.00–1.89]	0.045

*Close friends*				
0	Ref.		Ref.	
≥1	1.32 [1.07–1.63]	0.013	1.49 [1.17–1.89]	0.002

*Suicidal ideation*				
No	Ref.		Ref.	
Yes	2.5 [1.89–3.33]	0.000	1.58 [1.00–2.48]	0.05

*Suicidal planning*				
No	Ref.		Ref.	
Yes	1.85 [1.44–2.38]	0.00	1.02 [0.75–1.56]	0.620

*Suicidal attempt*				
No	Ref.		Ref.	
Yes	3.03 [2.17–4.17	0.000	1.88 [1.29–2.72]	0.001

*Truancy*				
No	Ref.		Ref.	
Yes	2.03 [1.69–2.44]	0.000	1.66 [1.31–2.09]	0.000

*Amphetamine*				
No	Ref.		Ref.	
Yes	7.14 [4.17–12.50]	0.000	2.95[1.46–5.95]	0.004

*Current drinker (alcohol)*				
No	Ref.		Ref.	
Yes	2.50 [1.72–3.57]	0.000	1.19 [0.84–1.69]	0.316

*Current smoker*				
No	Ref.		Ref.	
Yes	8.68[4.54–16.67]	0.000	2.64 [1.22–5.69]	0.015

^*∗*^Significant at *p* < 0.05; AOR = adjusted for all factors which appear in table; goodness of fit = *F* (9,17) = 0.51; *p* = 0.8495.

## Data Availability

Data for this study were sourced from WHO Global School Health Survey and are available at http://www.who.int/chp/gshs/en/.

## Additional Points

*Strengths and Limitations of the Study.* This study is from a representative sample data from the 2012 Global School-Based Student Health Survey (GSHS) and makes room for generalizability of study findings across Ghana. Also, observations with complete dataset meeting the study criteria were large. However, data/information was obtained from a cross-sectional study design, limiting inferences of causations. Furthermore, data were obtained retrospectively, introducing a high recall bias since participants self-reported. Recall bias has significant effect on coefficient estimates and overall significant testing; therefore, interpretations/use of the findings should be done cautiously. Also, strength of the approach thus employed logistic regression as a result of the dichotomous nature of the recategorized dependent variable, which poses a limitation to this current study. We recommend future studies could employ robust precise informing analysis such as count analysis of serious injuries (Poisson analysis to provide more precise risk ratios) without recategorization of the outcome variable. In addition, several factors such as hunger were estimated based on proxy measures, which limits general extrapolations of study findings.
